# Use of the Tei Index in the Conservative Management of TRAP Sequence Pregnancies Diagnosed during the Periviable Period: A Case Series

**DOI:** 10.1155/2018/2521797

**Published:** 2018-03-22

**Authors:** Kristina Martimucci, Theresa Kuhn, Robyn Bilinski, Jesus Alvarez-Perez

**Affiliations:** ^1^Rutgers New Jersey Medical School, Department of Obstetrics, Gynecology and Women's Health, Newark, NJ 07301, USA; ^2^Hackensack University Medical Center, Department of Obstetrics and Gynecology, Hackensack, NJ 07601, USA

## Abstract

Twin Reverse Arterial Perfusion (TRAP) Sequence is a rare complication of monochorionic pregnancies. Without intervention, the viable pump twin in a case of TRAP Sequence may develop high output cardiac failure leading to an intrauterine fetal demise. We present 3 cases of TRAP Sequence pregnancy diagnosed during the second or third trimesters of pregnancy. There are minimal sonographic tools for the guidance of a fetal therapeutic interventional procedure during the second trimester or timing of delivery during the third trimester to reduce morbidity and mortality of a viable fetus. Tei index may be a useful sonographic tool in the management of TRAP Sequence during the second or third trimester of pregnancy.

## 1. Introduction

TRAP Sequence, frequently referred to as acardiac twin syndrome, is a rare complication of monochorionic twin gestation with an incidence of 1/30000–1/35000 deliveries or 1% of all monochorionic pregnancies [1]. Aberrant placental vasculature connections of arterial-to-arterial and/or venous-to-venous shunting occur between twins resulting in a high-pressure gradient. This system creates a pathway in which the pump twin constantly pumps blood, not only throughout its own circulatory system, but also to support the acardiac twin.

Deoxygenated blood is pumped into the acardiac twin via the umbilical arteries. Preferential shunting into the iliac blood vessels occurs perfusing the lower body and hindering the growth of the upper body [2]. The degree of perfusion will determine the extent of the aberrant formation of the acardiac twin. The acardiac twin nomenclature includes acardiac acephalus (no identifiable growth of the head), acardiac myelacephalus (partially developed head and limbs), acardiac amorphous (no identifiable structures), acardiac anceps (well-developed lower extremities and abdomen with a poorly developed head), and acardius acornus (head connected to placenta) [3].

Diagnosis of a TRAP Sequence pregnancy may readily be made in early first-trimester ultrasounds (11–14 weeks) or during the time of the detailed anatomy ultrasound (18–23 weeks) [4]. The data for the management is clear when a diagnosis of TRAP Sequence is diagnosed in the first trimester. Timed interventions confer a higher survival rate of the pump twin compared to conservative approach [5] and include endoscopic ligation of the abnormal vasculature, laser coagulation/cauterization of the umbilical cord, or radiofrequency ablation [6]. Conservative management of a TRAP Sequence pregnancy results in a 50%–75% mortality of the pump twin. The mortality rate increases as the size of the acardiac twin increases [1]. The expected weight of the acardiac twin cannot be estimated using the classic Hadlock formula and is instead calculated via the following formula: (1.2 × longitudinal axis^∧^2) – (1.7 × longitudinal axis) [7]. Unfortunately, the majority of the first-trimester obstetrical ultrasounds between 11 and 14 weeks of gestation are being performed in the General Obstetricians Office or a General Radiology Office and not in a specialized Maternal Fetal Medicine Unit. Many early anatomical fetal anomalies and cases like TRAP Sequence are missed early and identified during the anatomy ultrasound.

There is limited data in regard to the management of TRAP Sequence pregnancies made around the periviable period or in patients that opt for conservative management. Prognostic factors that could theoretically help guide management decisions are also lacking; however they include the size of the acardiac twin and the apparent condition of the pump twin [4]. The myocardial performance index (Tei index) is an echocardiographic/Doppler index that combines systolic and diastolic function and is calculated as isovolumic relaxation time plus isovolumic contraction time divided by ejection time. The Tei index has been used to study heart failure in adults, and it can also be used to quantitatively evaluate congestive heart failure in fetuses with hydrops fetalis. This case series suggests that the Tei index may be a useful time sensitive tool in the management of a pregnancy complicated by TRAP Sequence.

## 2. Case Presentations

### 2.1. Case  1

The patient is a 25-year-old G1P0 initially presenting to our L&D triage with a 28 weeks and 2 days of monochorionic diamniotic twin gestation with a diagnosis of threatened preterm labor. Her antenatal history included demise of twin B occurring at 11 w 6 d and an abnormal QUAD Screen with a 1 : 6 risk of open spina bifida. The last sonographic evaluation during the pregnancy was done at an outside institution reporting a viable 20 w 5 d fetus with an EFW of 365 g and a demise twin B with measurements consistent with a 13 w 4 d gestation.

An ultrasound performed upon admission confirmed the dating of the pregnancy and a diagnosis of TRAP Sequence was made. The ultrasound findings were the following: twin A (pump twin), an EFW of 953 g (27th percentile) was obtained, the Ductus Venosus (DV) and Middle Cerebral Artery (MCA) Doppler flows values were normal, an umbilical artery (UA) Doppler S/D ratio of 5.0 (>4.25 is greater than the 95% ile), and the left sided Tei index was 0.4. There was no evidence of cardiomegaly, pericardial effusion, or polyhydramnios. We were unable to ascertain an expected weight for twin B (the acardiac twin). A management plan was made for surveillance three times weekly with Biophysical Profile (BPP), UA, MCA, DV Doppler flows, and Tei index.

At 29 w 2 d, the UA S/D ratio was 4.53, the MCA-PSV was 44.0 cm/second and PI was 1.7, the DV did not have an absent or an abnormal a-wave, the BPP 6/10 (−2 for breathing and −2 for minimal variability on the NST), and the left sided Tei index was 0.6 with evidence of mild cardiomegaly with mild tricuspid regurgitation. A decision for delivery was made.

**Figure 1:**
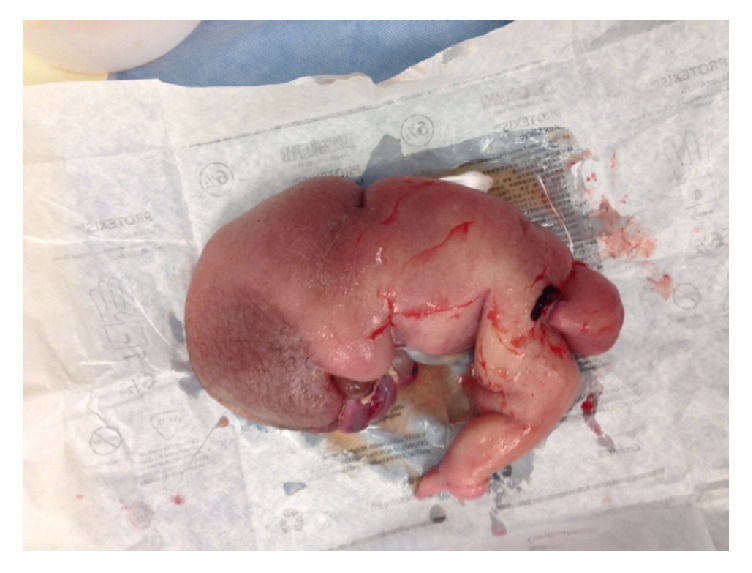
Case # 1: acardiac twin. Weight 1000 g.

**Figure 2:**
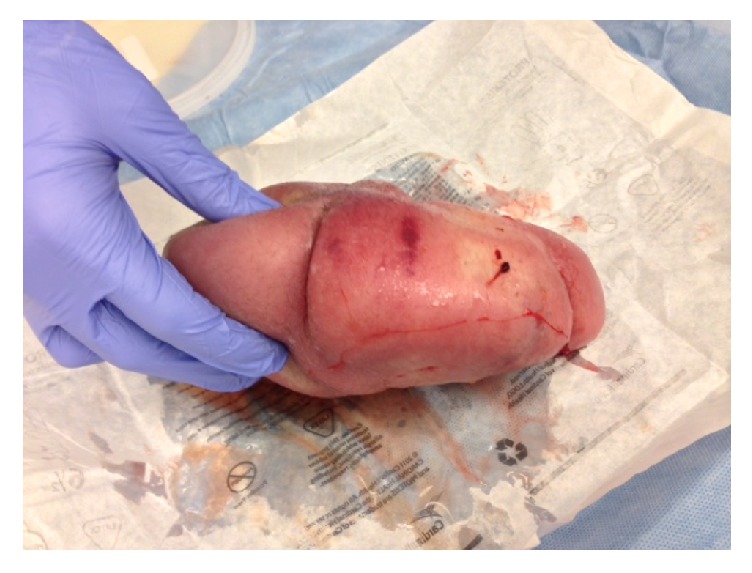
Case # 1: acardiac twin. View from above.

Twin A had an APGAR score of 7.8 with a neonatal weight of 1000 g. The acardiac twin had a weight of 1000 g as well (see Figures 1 and 2). The initial cord gases showed a pH of 7.14. The maternal postoperative course was unremarkable and she was discharged home on postoperative day three.

The pump twin's neonatal course was complicated by RDS requiring intubation with surfactant administration. Neonatal echocardiogram confirmed mild left ventricular dilation and a small PDA. The infant was discharged home in stable condition 7 weeks after delivery.

### 2.2. Case  2

The patient is a 38-year-old G13P10 in which a late diagnosis of TRAP Sequence was made at 28 weeks. After the diagnosis was made, the patient's care was transferred to our institution.

Our initial ultrasound evaluation showed the pump twin to have a BPP of 8/8, normal UA, DV, and MCA Doppler flows. The left side of Tei index was 0.42. A sonogram for antenatal surveillance was done twice weekly and the pump twin remained stable until 32 w 6 d of gestation.

At 32 w 6 d of gestation the sonographic findings were the following: the BPP was 6/8 (−2 for breathing), UA Doppler S/D ratio was 5.7, MCA-PI was 1.3 and the PSV was 60 cm/second, the DV Doppler flow was normal, and the left Tei was 0.64 with both moderate tricuspid and mitral valve regurgitation. Left ventricular hypertrophy was suspected with the ventricular walls measurements being 0.6 mm. Polyhydramnios were identified with an AFI of 30. Decision for delivery was made due to the abnormal BPP and finding of ventricular hypertrophy.

**Figure 3:**
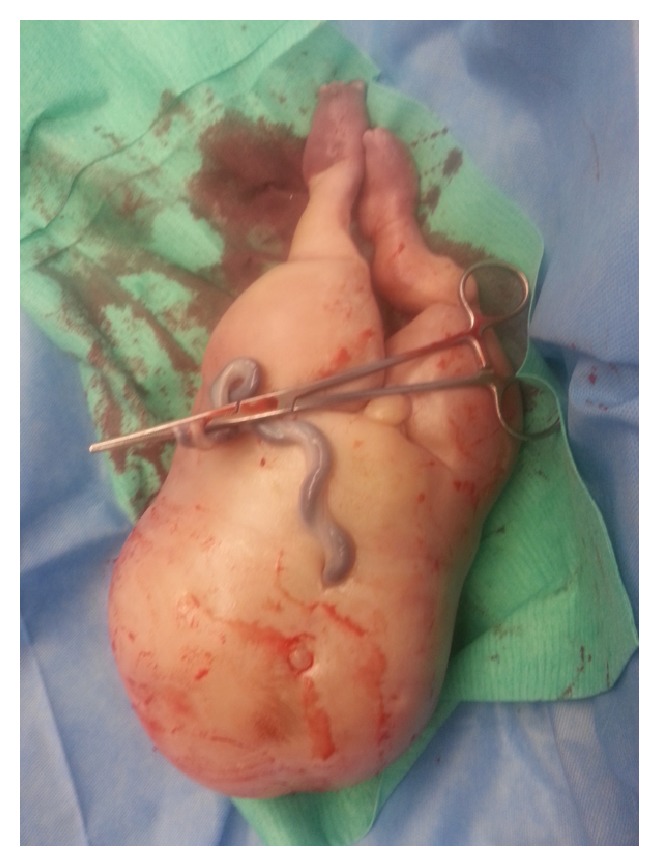
Case # 2: acardiac twin. Weight 995 g.

A cesarean delivery was done and twin A (the acardiac twin) was delivered followed by twin B (pump twin). APGARs of 8.9 were given and the initial cord gases showed a pH of 7.2. The neonatal weight for twin B was 2480 grams and the weight of the acardiac twin was 995 grams (see Figure 3).

Neonatal course was complicated by mild respiratory distress managed only with C-PAP and supportive treatment. A neonatal echocardiogram showed a large PDA, a small ASD, and a left ventricular dilation due to intrauterine volume overload.

### 2.3. Case  3

The patient is a 22-year-old G2P0, late registrant for prenatal care referred for an anatomy scan at 22 weeks of gestation. A TRAP Sequence was diagnosed and the following findings were identified: the UA S/D ratio was 6.2, the DV Doppler flow showed an abnormal resistance but no abnormal a-wave, the MCA-PI was 2.0, and the MCA-PSV was 31. The left sided Tei index was 0.55.

We explained the pathophysiology of a TRAP Sequence in detail. Since we suspected cardiac dysfunction of the pump twin, an emergent intrauterine radiofrequency ablation of the cord for the acardiac twin was offered but the patient declined and opted for a second opinion.

The patient presented 4 days later with decreased fetal movement and an ultrasound confirmed a fetal demise of the pump twin.

Induction of labor with misoprostol was started and 16 hours later the fetuses were delivered. The pump twin had an EFW of 452 grams and the acardiac twin weighed 388 grams. The patient was discharged on postpartum day 1.

## 3. Discussion

The management of TRAP Sequence has been controversial due to the paucity of literature with many proposed methods loosely based on gestational age. Surgical management in a previable pregnancy is largely managed by interrupting the aberrant vasculature of the pump twin towards the acardiac twin and designed to arrest the reverse flow from the pump twin to the acardiac twin, improving the prognosis of the pump twin^2^. For this reason, it is vital for the diagnosis to be made in the first trimester or early second trimester when these interventions can take place. In 2003, a review by Tan and Sepulveda [8]. stated that radiofrequency ablation is superior to cord occlusion procedures. Ablation has been associated with a delayed gestational age at delivery and a lower technical failure rate. Survival rate of ablation ranges between 86% and 94% when performed between 16 and 24 weeks in comparison to other techniques like fetoscopic laser coagulation having a reported survival rate of 80% [8–11].

There is limited data in regard to the conservative management of TRAP Sequence for those patients diagnosed after 24 weeks of gestational age. Dashe et al. presented a study of ten patients diagnosed between 18 and 24 weeks of gestational age who were conservatively managed using serial sonography. In their study, 90% of babies were delivered between 29 and 38 weeks of gestational age and in four cases, Doppler showed a reduction of blood flow to the acardiac twin [12]. Pepe reported a case where weekly b-mode ultrasound and Color Doppler were used to assess the well-being of the pump fetus^2^ in which pregnancy resulted in normal appearing female at 36 weeks of gestation. Harjai et al. studied echocardiography in the pump twin in nine pregnancies complicated by TRAP Sequence and determined that abnormal left ventricular shortening fraction was associated with a worse outcome [6]. Furthermore, Dashe et al. studied six pregnancies complicated with TRAP Sequence between 16 to 34 weeks and found that Doppler studies with a large umbilical artery resistive index differences (defined as the peak systolic velocity minus the end diastolic velocity divided by the peak systolic velocity) between the pump and acardiac twins resulted in a good prognosis, but a poor prognosis when the umbilical artery resistive index difference was small [12].

When untreated, TRAP Sequence may result in a high output cardiac failure of the pump twin. We used Tei index, myocardial performance index, in the management of pregnancies complicated with TRAP Sequence. A commonality between all three cases was the increased Tei index in the compromised pump twin. The Tei index has been used to study heart failure in adults with prolong indices predicting poor outcomes in patients having congestive heart failure and systolic dysfunction [6]. Falkensammer and colleagues used the Tei index to quantitatively evaluate congestive heart failure in fetuses with hydrops fetalis. They noted that Tei index is useful in the periodic assessment of the cardiac status in a fetus with hydrops [13]. Given the high likelihood of cardiac decompensation and risk of cardiovascular collapse of the pump twin fetus, our management was guided by the antepartum surveillance testing (Biophysical Profile and Doppler Flow studies) and the use of the Tei index.

The normal value for a Tei index is less than 0.4 [13, 14]. When the value of the Tei index is above 0.5, the fetus most likely has developed myocardial dysfunction and may be at risk of metabolic acidosis and/or fetal demise.

Tei index is used in the antenatal management of twins with twin-to-twin transfusion syndrome, fetal hydrops, or fetal myocardial dysfunction [13–15]. We propose that Tei index may be a useful sonographic tool for the guidance of intrauterine intervention, conservative management, or delivery in cases of TRAP Sequence pregnancies when the diagnosis of TRAP Sequence is first made during the periviable gestational period. Further studies are warranted.

## Additional Points

*Established Facts*. (1) Conservative management of a TRAP Sequence pregnancy results in a 50%–75% mortality of the pump twin. (2) A previability diagnosis and management of TRAP Sequence may include endoscopic ligation of the abnormal vasculature, laser coagulation/cauterization of the umbilical cord, and radiofrequency ablation.* Novel Insights.* (1) Myocardial Performance Index (Tei index) is a useful time sensitive tool in the management of a pregnancy complicated by TRAP Sequence. (2) When the value of the Tei index is above 0.5, the fetus most likely has developed myocardial dysfunction and may be at risk of metabolic acidosis and/or fetal demise.
